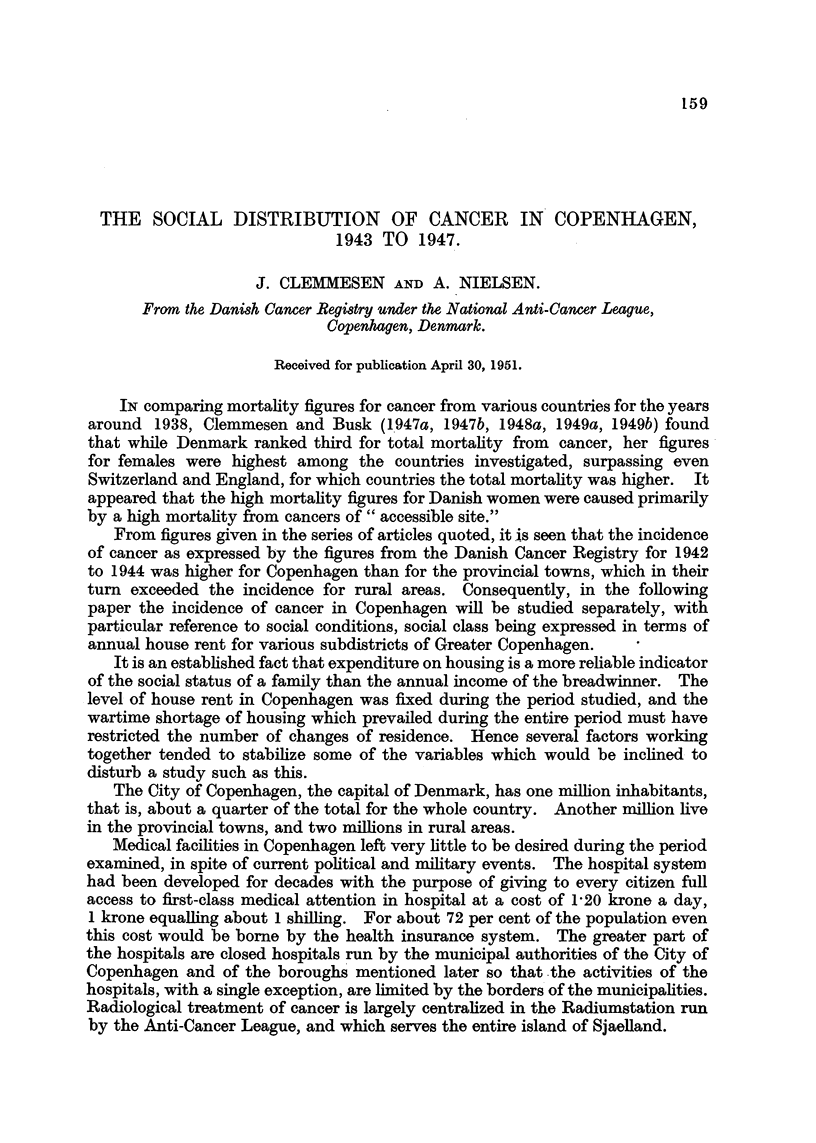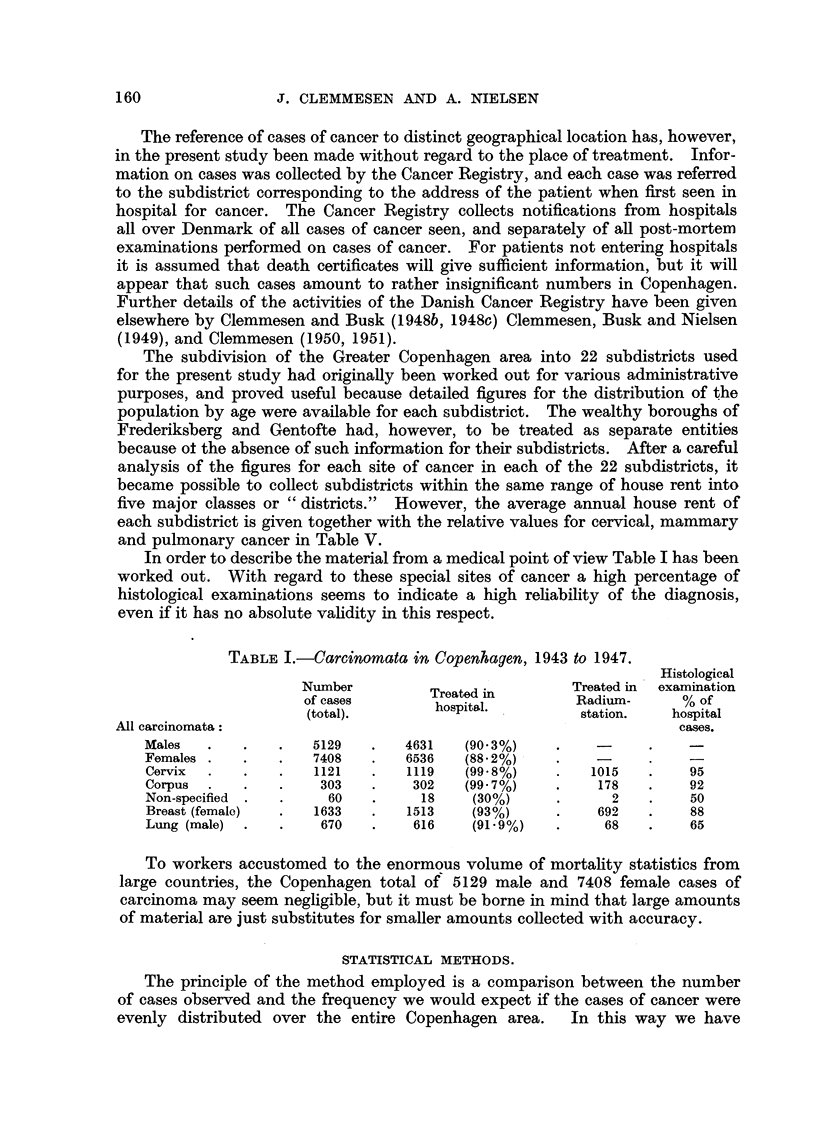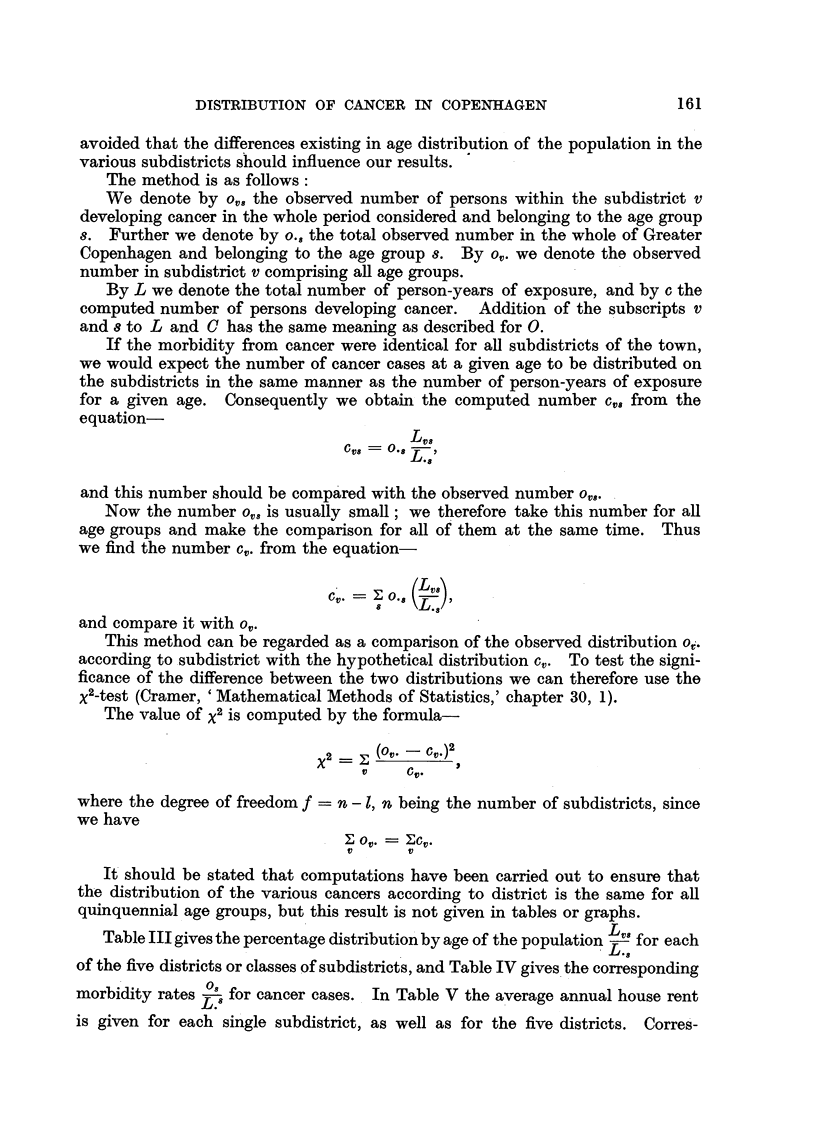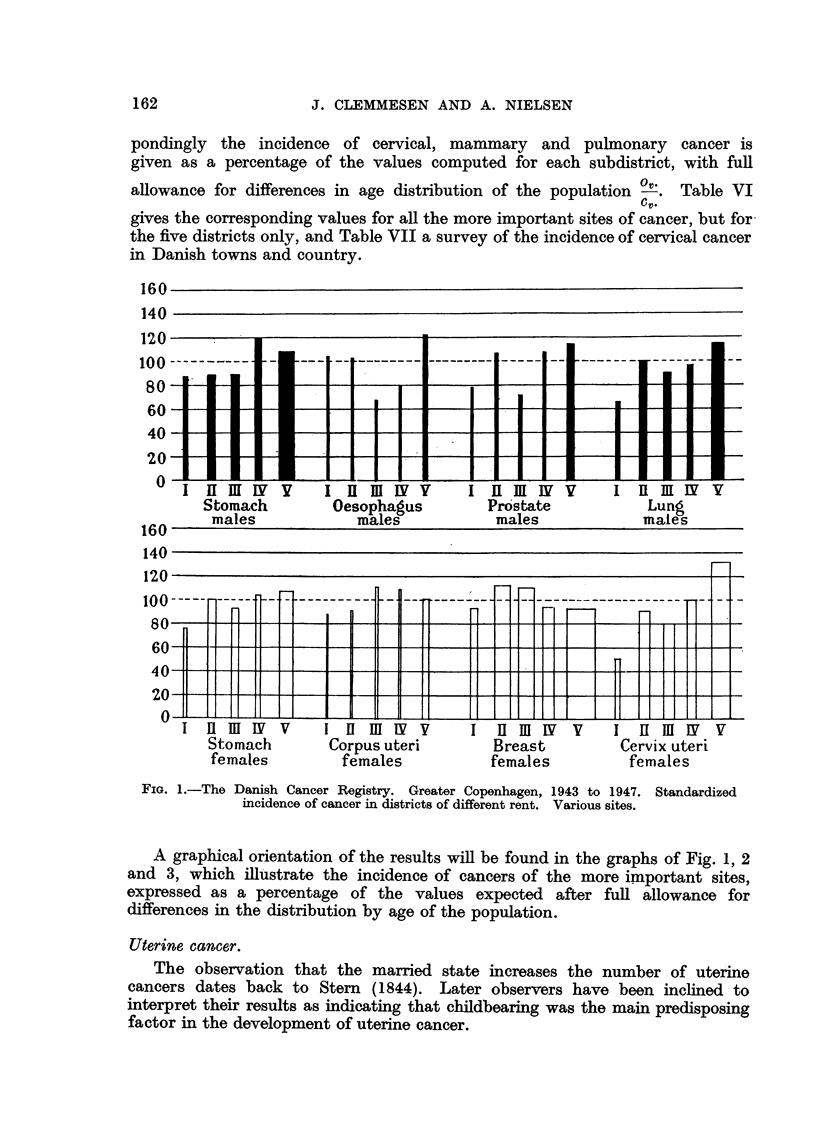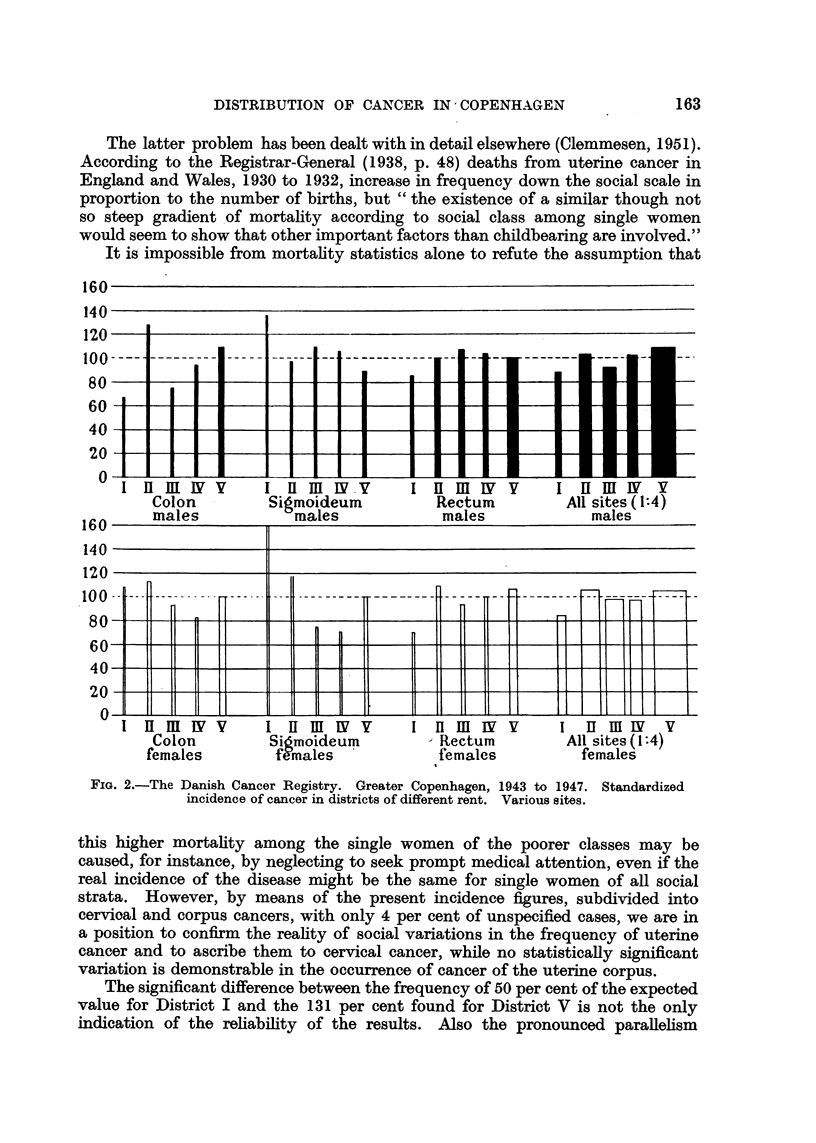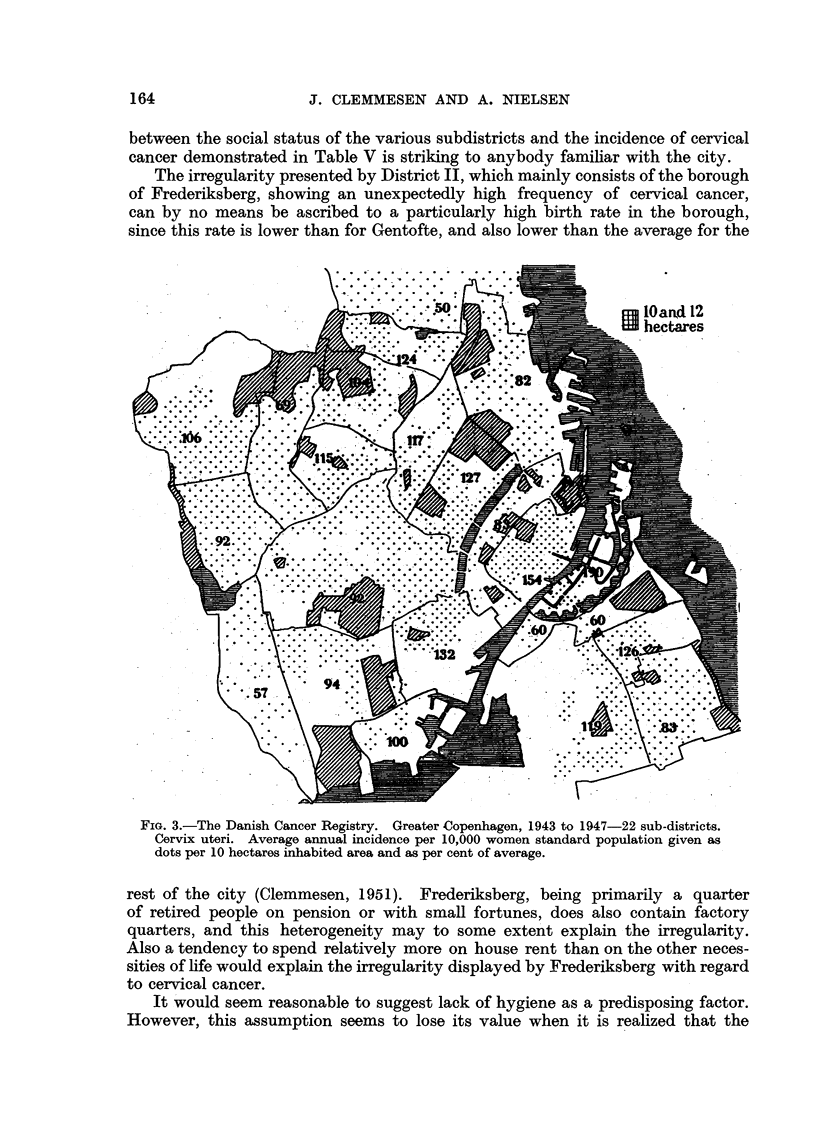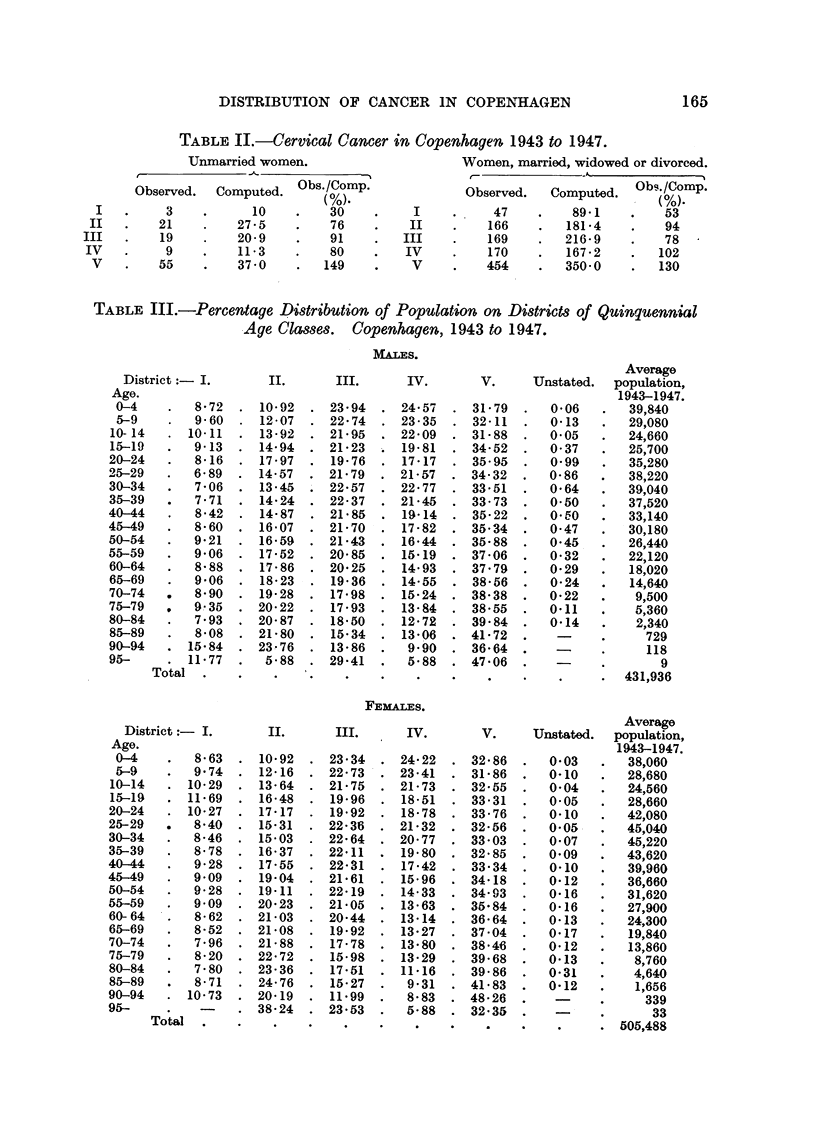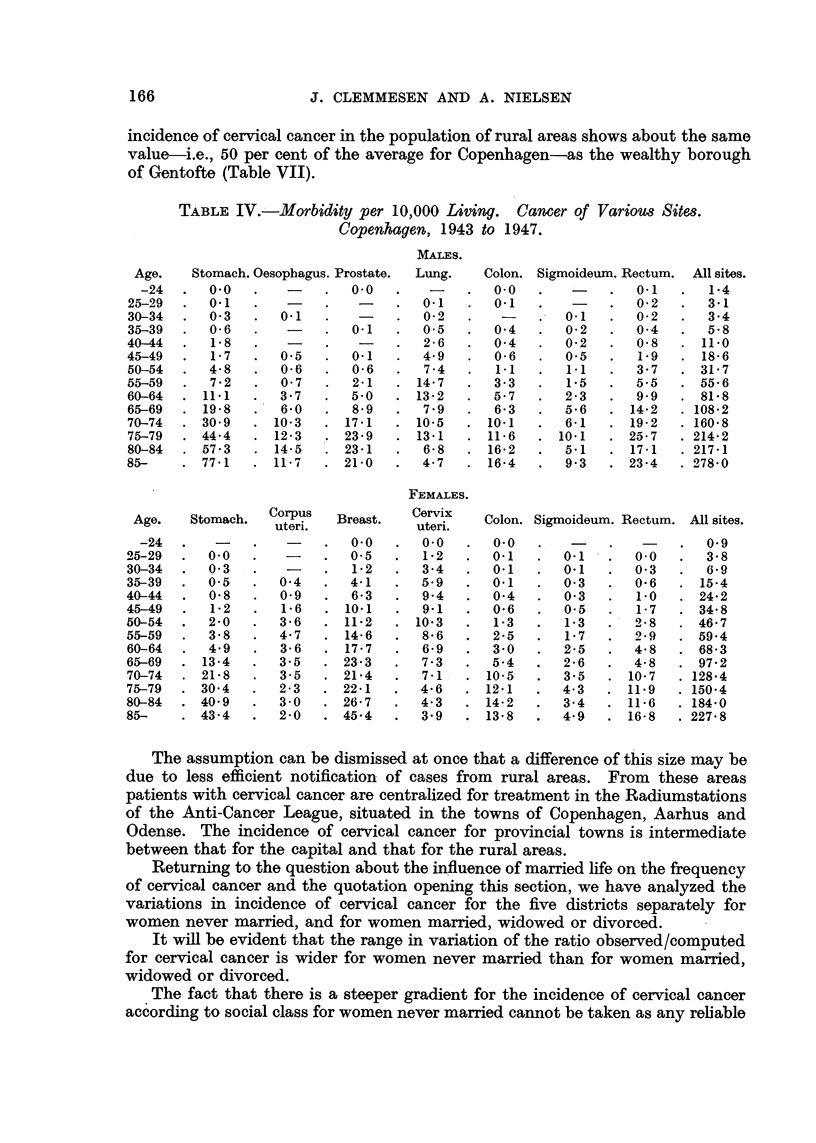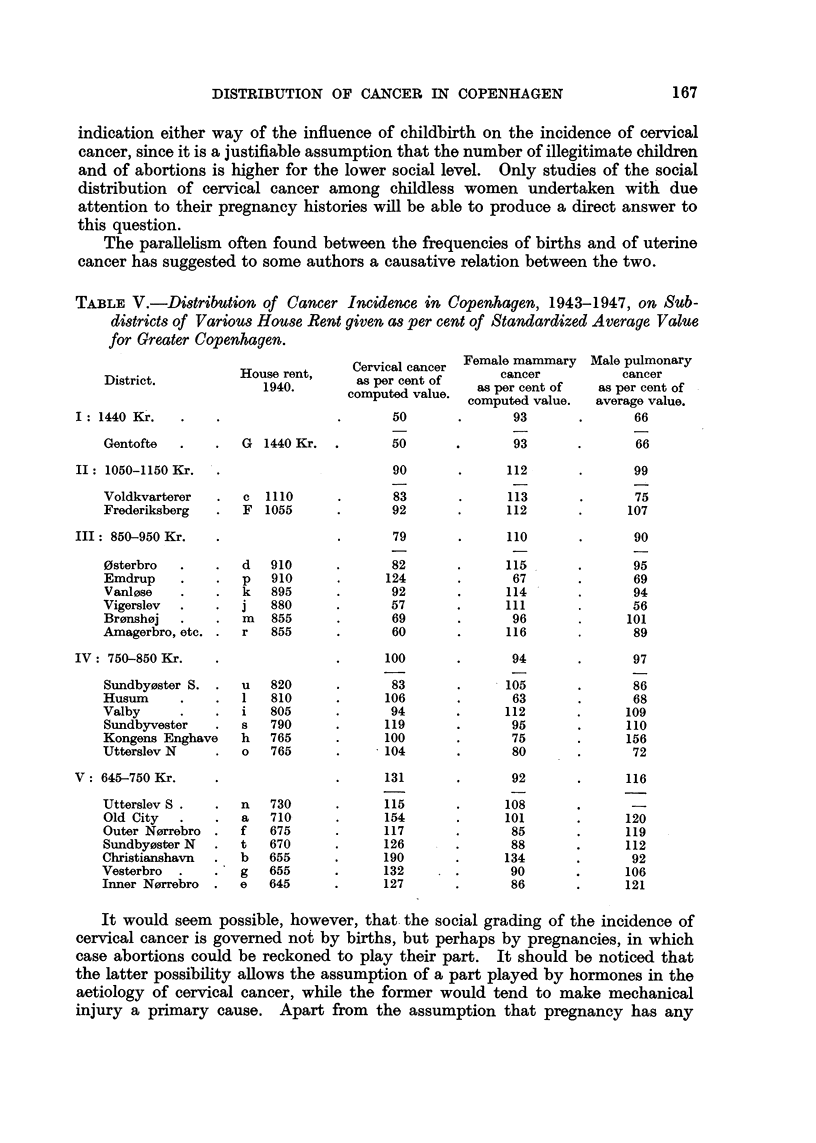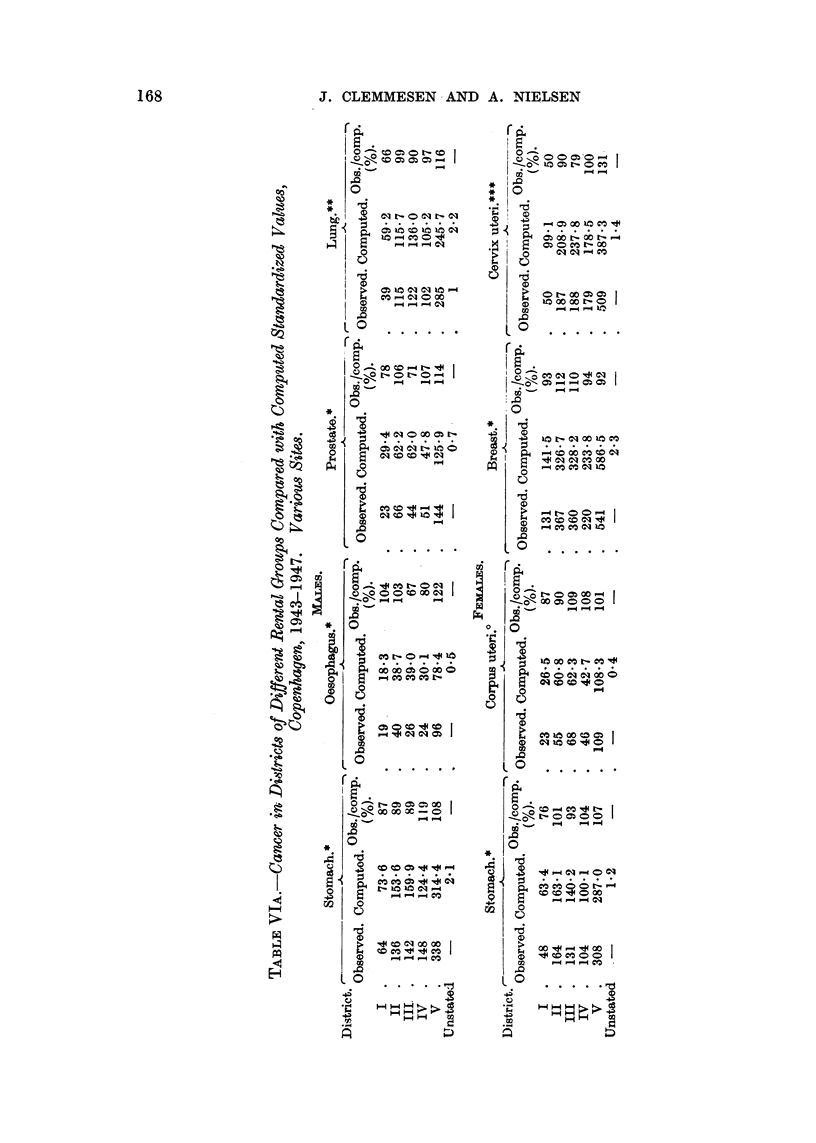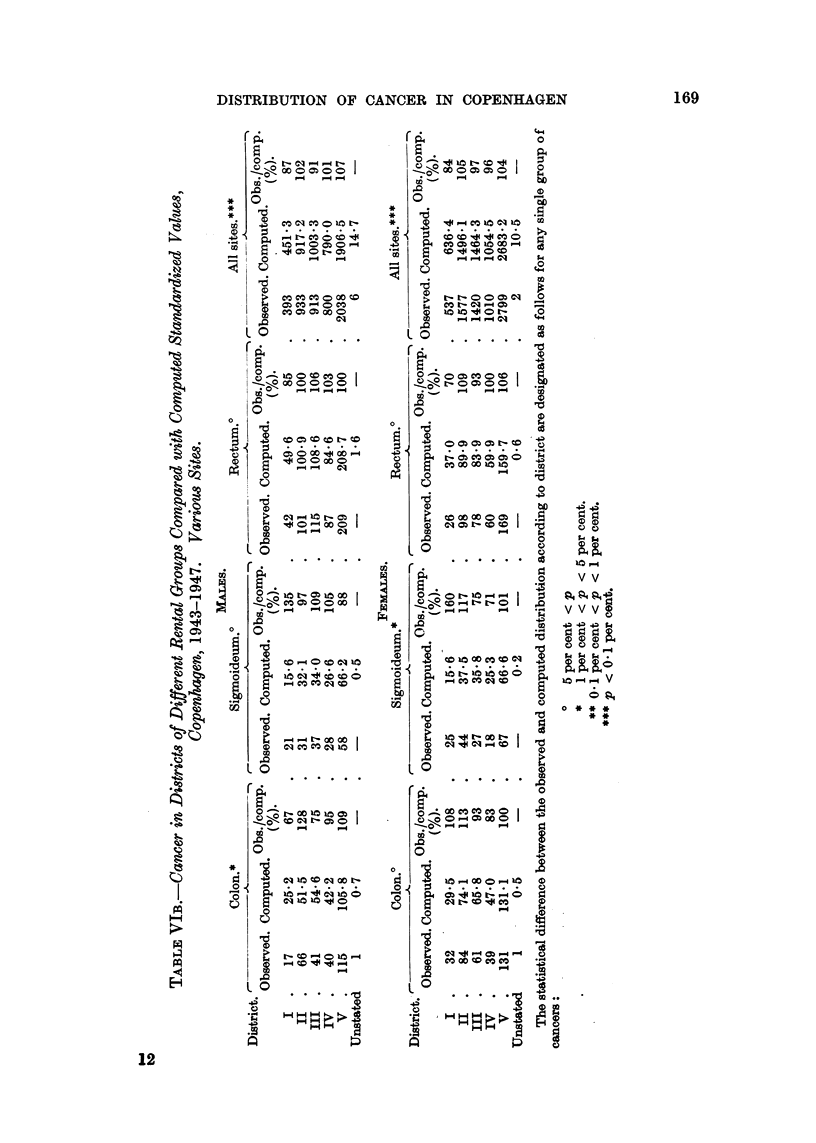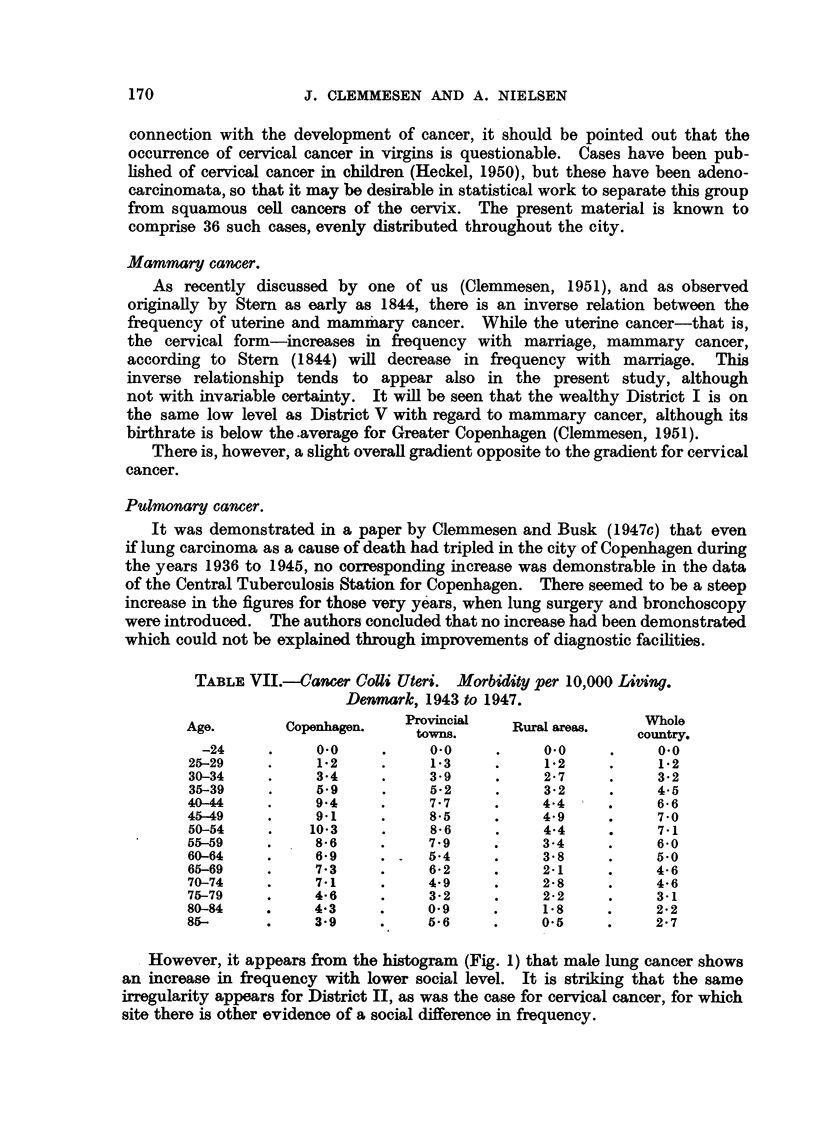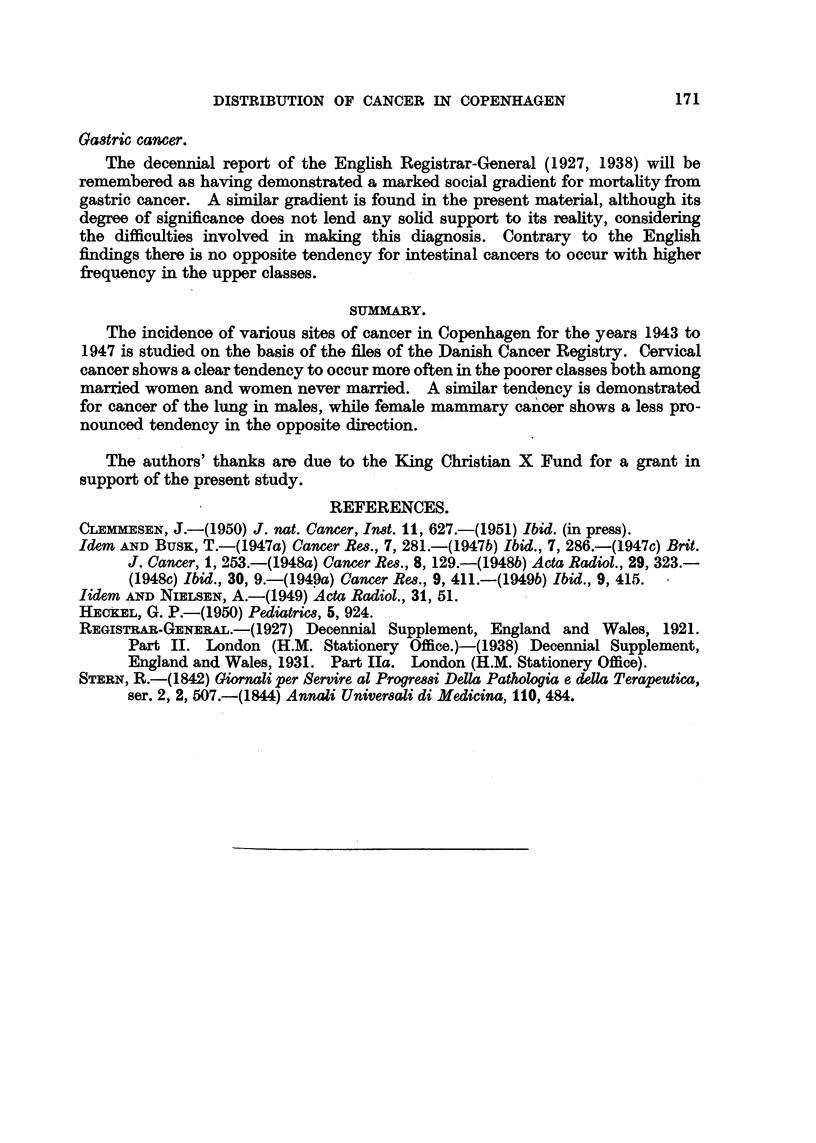# The Social Distribution of Cancer in Copenhagen, 1943 to 1947

**DOI:** 10.1038/bjc.1951.17

**Published:** 1951-06

**Authors:** J. Clemmesen, A. Nielsen


### THE SOCIAL DISTRIBUTION OF CANCER IN COPENHAGEN, 1943 TO 1947.

J. CLEMMESEN AND A. NIELSEN.

*From the Danish Cancer Registry under the National Anti-Cancer League, Copenhagen, Denmark.*

Received for publication April 30, 1951.

In comparing mortality figures for cancer from various countries for the years around 1938, Clemmesen and Busk (1947*a*, 1947*b*, 1948*a*, 1949*a*, 1949*b*) found that while Denmark ranked third for total mortality from cancer, her figures for females were highest among the countries investigated, surpassing even Switzerland and England, for which countries the total mortality was higher. It appeared that the high mortality figures for Danish women were caused primarily by a high mortality from cancers of " accessible site."

From figures given in the series of articles quoted, it is seen that the incidence of cancer as expressed by the figures from the Danish Cancer Registry for 1942 to 1944 was higher for Copenhagen than for the provincial towns, which in their turn exceeded the incidence for rural areas. Consequently, in the following paper the incidence of cancer in Copenhagen will be studied separately, with particular reference to social conditions, social class being expressed in terms of annual house rent for various subdistricts of Greater Copenhagen.

It is an established fact that expenditure on housing is a more reliable indicator of the social status of a family than the annual income of the breadwinner. The level of house rent in Copenhagen was fixed during the period studied, and the wartime shortage of housing which prevailed during the entire period must have restricted the number of changes of residence. Hence several factors working together tended to stabilize some of the variables which would be inclined to disturb a study such as this.

The City of Copenhagen, the capital of Denmark, has one million inhabitants, that is, about a quarter of the total for the whole country. Another million live in the provincial towns, and two millions in rural areas.

Medical facilities in Copenhagen left very little to be desired during the period examined, in spite of current political and military events. The hospital system had been developed for decades with the purpose of giving to every citizen full access to first-class medical attention in hospital at a cost of 1·20 krone a day, 1 krone equalling about 1 shilling. For about 72 per cent of the population even this cost would be borne by the health insurance system. The greater part of the hospitals are closed hospitals run by the municipal authorities of the City of Copenhagen and of the boroughs mentioned later so that the activities of the hospitals, with a single exception, are limited by the borders of the municipalities. Radiological treatment of cancer is largely centralized in the Radiumstation run by the Anti-Cancer League, and which serves the entire island of Sjaelland.

The reference of cases of cancer to distinct geographical location has, however, in the present study been made without regard to the place of treatment. Information on cases was collected by the Cancer Registry, and each case was referred to the subdistrict corresponding to the address of the patient when first seen in hospital for cancer. The Cancer Registry collects notifications from hospitals all over Denmark of all cases of cancer seen, and separately of all post-mortem examinations performed on cases of cancer. For patients not entering hospitals it is assumed that death certificates will give sufficient information, but it will appear that such cases amount to rather insignificant numbers in Copenhagen. Further details of the activities of the Danish Cancer Registry have been given elsewhere by Clemmesen and Busk (1948*b*, 1948*c*) Clemmesen, Busk and Nielsen (1949), and Clemmesen (1950, 1951).

The subdivision of the Greater Copenhagen area into 22 subdistricts used for the present study had originally been worked out for various administrative purposes, and proved useful because detailed figures for the distribution of the population by age were available for each subdistrict. The wealthy boroughs of Frederiksberg and Gentofte had, however, to be treated as separate entities because of the absence of such information for their subdistricts. After a careful analysis of the figures for each site of cancer in each of the 22 subdistricts, it became possible to collect subdistricts within the same range of house rent into five major classes or " districts." However, the average annual house rent of each subdistrict is given together with the relative values for cervical, mammary and pulmonary cancer in Table V.

In order to describe the material from a medical point of view Table I has been worked out. With regard to these special sites of cancer a high percentage of histological examinations seems to indicate a high reliability of the diagnosis, even if it has no absolute validity in this respect.

TABLE I.—*Carcinomata in Copenhagen*, 1943 *to* 1947.

| All carcinomata : | Number of cases (total). | Treated in hospital. | | Treated in Radium-station. | Histological examination % of hospital cases. |
|---|---|---|---|---|---|
| Males | 5129 | 4631 | (90·3%) | — | — |
| Females | 7408 | 6536 | (88·2%) | — | — |
| Cervix | 1121 | 1119 | (99·8%) | 1015 | 95 |
| Corpus | 303 | 302 | (99·7%) | 178 | 92 |
| Non-specified | 60 | 18 | (30%) | 2 | 50 |
| Breast (female) | 1633 | 1513 | (93%) | 692 | 88 |
| Lung (male) | 670 | 616 | (91·9%) | 68 | 65 |

To workers accustomed to the enormous volume of mortality statistics from large countries, the Copenhagen total of 5129 male and 7408 female cases of carcinoma may seem negligible, but it must be borne in mind that large amounts of material are just substitutes for smaller amounts collected with accuracy.

STATISTICAL METHODS.

The principle of the method employed is a comparison between the number of cases observed and the frequency we would expect if the cases of cancer were evenly distributed over the entire Copenhagen area. In this way we have avoided that the differences existing in age distribution of the population in the various subdistricts should influence our results.

The method is as follows :

We denote by $o_{vs}$ the observed number of persons within the subdistrict $v$ developing cancer in the whole period considered and belonging to the age group $s$. Further we denote by $o_{.s}$ the total observed number in the whole of Greater Copenhagen and belonging to the age group $s$. By $o_{v.}$ we denote the observed number in subdistrict $v$ comprising all age groups.

By $L$ we denote the total number of person-years of exposure, and by $c$ the computed number of persons developing cancer. Addition of the subscripts $v$ and $s$ to $L$ and $C$ has the same meaning as described for $O$.

If the morbidity from cancer were identical for all subdistricts of the town, we would expect the number of cancer cases at a given age to be distributed on the subdistricts in the same manner as the number of person-years of exposure for a given age. Consequently we obtain the computed number $c_{vs}$ from the equation—

$$c_{vs} = o_{.s} \frac{L_{vs}}{L_{.s}},$$

and this number should be compared with the observed number $o_{vs}$.

Now the number $o_{vs}$ is usually small ; we therefore take this number for all age groups and make the comparison for all of them at the same time. Thus we find the number $c_{v.}$ from the equation—

$$c_{v.} = \sum_{s} o_{.s} \left(\frac{L_{vs}}{L_{.s}}\right),$$

and compare it with $o_{v.}$

This method can be regarded as a comparison of the observed distribution $o_{v.}$ according to subdistrict with the hypothetical distribution $c_{v.}$ To test the significance of the difference between the two distributions we can therefore use the $\chi^2$-test (Cramer, ' Mathematical Methods of Statistics,' chapter 30, 1).

The value of $\chi^2$ is computed by the formula—

$$\chi^2 = \sum_{v} \frac{(o_{v.} - c_{v.})^2}{c_{v.}},$$

where the degree of freedom $f = n - l$, $n$ being the number of subdistricts, since we have

$$\sum_{v} o_{v.} = \sum_{v} c_{v.}$$

It should be stated that computations have been carried out to ensure that the distribution of the various cancers according to district is the same for all quinquennial age groups, but this result is not given in tables or graphs.

Table III gives the percentage distribution by age of the population $\frac{L_{vs}}{L_{.s}}$ for each of the five districts or classes of subdistricts, and Table IV gives the corresponding morbidity rates $\frac{o_{s}}{L_{.s}}$ for cancer cases. In Table V the average annual house rent is given for each single subdistrict, as well as for the five districts. Correspondingly the incidence of cervical, mammary and pulmonary cancer is given as a percentage of the values computed for each subdistrict, with full allowance for differences in age distribution of the population $\frac{O_v}{C_v}$. Table VI gives the corresponding values for all the more important sites of cancer, but for the five districts only, and Table VII a survey of the incidence of cervical cancer in Danish towns and country.

FIG. 1.—The Danish Cancer Registry. Greater Copenhagen, 1943 to 1947. Standardized incidence of cancer in districts of different rent. Various sites.

A graphical orientation of the results will be found in the graphs of Fig. 1, 2 and 3, which illustrate the incidence of cancers of the more important sites, expressed as a percentage of the values expected after full allowance for differences in the distribution by age of the population.

*Uterine cancer.*

The observation that the married state increases the number of uterine cancers dates back to Stern (1844). Later observers have been inclined to interpret their results as indicating that childbearing was the main predisposing factor in the development of uterine cancer.

The latter problem has been dealt with in detail elsewhere (Clemmesen, 1951). According to the Registrar-General (1938, p. 48) deaths from uterine cancer in England and Wales, 1930 to 1932, increase in frequency down the social scale in proportion to the number of births, but " the existence of a similar though not so steep gradient of mortality according to social class among single women would seem to show that other important factors than childbearing are involved."

It is impossible from mortality statistics alone to refute the assumption that

FIG. 2.—The Danish Cancer Registry. Greater Copenhagen, 1943 to 1947. Standardized incidence of cancer in districts of different rent. Various sites.

this higher mortality among the single women of the poorer classes may be caused, for instance, by neglecting to seek prompt medical attention, even if the real incidence of the disease might be the same for single women of all social strata. However, by means of the present incidence figures, subdivided into cervical and corpus cancers, with only 4 per cent of unspecified cases, we are in a position to confirm the reality of social variations in the frequency of uterine cancer and to ascribe them to cervical cancer, while no statistically significant variation is demonstrable in the occurrence of cancer of the uterine corpus.

The significant difference between the frequency of 50 per cent of the expected value for District I and the 131 per cent found for District V is not the only indication of the reliability of the results. Also the pronounced parallelism between the social status of the various subdistricts and the incidence of cervical cancer demonstrated in Table V is striking to anybody familiar with the city.

The irregularity presented by District II, which mainly consists of the borough of Frederiksberg, showing an unexpectedly high frequency of cervical cancer, can by no means be ascribed to a particularly high birth rate in the borough, since this rate is lower than for Gentofte, and also lower than the average for the

FIG. 3.—The Danish Cancer Registry. Greater Copenhagen, 1943 to 1947—22 sub-districts. Cervix uteri. Average annual incidence per 10,000 women standard population given as dots per 10 hectares inhabited area and as per cent of average.

rest of the city (Clemmesen, 1951). Frederiksberg, being primarily a quarter of retired people on pension or with small fortunes, does also contain factory quarters, and this heterogeneity may to some extent explain the irregularity. Also a tendency to spend relatively more on house rent than on the other necessities of life would explain the irregularity displayed by Frederiksberg with regard to cervical cancer.

It would seem reasonable to suggest lack of hygiene as a predisposing factor. However, this assumption seems to lose its value when it is realized that the

TABLE II.—*Cervical Cancer in Copenhagen* 1943 *to* 1947.

| | Unmarried women. | | | | Women, married, widowed or divorced. | | |
|---|---|---|---|---|---|---|---|
| | Observed. | Computed. | Obs./Comp. (%). | | Observed. | Computed. | Obs./Comp. (%). |
| I | 3 | 10 | 30 | I | 47 | 89·1 | 53 |
| II | 21 | 27·5 | 76 | II | 166 | 181·4 | 94 |
| III | 19 | 20·9 | 91 | III | 169 | 216·9 | 78 |
| IV | 9 | 11·3 | 80 | IV | 170 | 167·2 | 102 |
| V | 55 | 37·0 | 149 | V | 454 | 350·0 | 130 |

TABLE III.—*Percentage Distribution of Population on Districts of Quinquennial Age Classes. Copenhagen,* 1943 *to* 1947.

MALES.

| District :— Age. | I. | II. | III. | IV. | V. | Unstated. | Average population, 1943–1947. |
|---|---|---|---|---|---|---|---|
| 0–4 | 8·72 | 10·92 | 23·94 | 24·57 | 31·79 | 0·06 | 39,840 |
| 5–9 | 9·60 | 12·07 | 22·74 | 23·35 | 32·11 | 0·13 | 29,080 |
| 10–14 | 10·11 | 13·92 | 21·95 | 22·09 | 31·88 | 0·05 | 24,660 |
| 15–19 | 9·13 | 14·94 | 21·23 | 19·81 | 34·52 | 0·37 | 25,700 |
| 20–24 | 8·16 | 17·97 | 19·76 | 17·17 | 35·95 | 0·99 | 35,280 |
| 25–29 | 6·89 | 14·57 | 21·79 | 21·57 | 34·32 | 0·86 | 38,220 |
| 30–34 | 7·06 | 13·45 | 22·57 | 22·77 | 33·51 | 0·64 | 39,040 |
| 35–39 | 7·71 | 14·24 | 22·37 | 21·45 | 33·73 | 0·50 | 37,520 |
| 40–44 | 8·42 | 14·87 | 21·85 | 19·14 | 35·22 | 0·50 | 33,140 |
| 45–49 | 8·60 | 16·07 | 21·70 | 17·82 | 35·34 | 0·47 | 30,180 |
| 50–54 | 9·21 | 16·59 | 21·43 | 16·44 | 35·88 | 0·45 | 26,440 |
| 55–59 | 9·06 | 17·52 | 20·85 | 15·19 | 37·06 | 0·32 | 22,120 |
| 60–64 | 8·88 | 17·86 | 20·25 | 14·93 | 37·79 | 0·29 | 18,020 |
| 65–69 | 9·06 | 18·23 | 19·36 | 14·55 | 38·56 | 0·24 | 14,640 |
| 70–74 | 8·90 | 19·28 | 17·98 | 15·24 | 38·38 | 0·22 | 9,500 |
| 75–79 | 9·35 | 20·22 | 17·93 | 13·84 | 38·55 | 0·11 | 5,360 |
| 80–84 | 7·93 | 20·87 | 18·50 | 12·72 | 39·84 | 0·14 | 2,340 |
| 85–89 | 8·08 | 21·80 | 15·34 | 13·06 | 41·72 | — | 729 |
| 90–94 | 15·84 | 23·76 | 13·86 | 9·90 | 36·64 | — | 118 |
| 95– | 11·77 | 5·88 | 29·41 | 5·88 | 47·06 | — | 9 |
| Total | | | | | | | 431,936 |

FEMALES.

| District :— Age. | I. | II. | III. | IV. | V. | Unstated. | Average population, 1943–1947. |
|---|---|---|---|---|---|---|---|
| 0–4 | 8·63 | 10·92 | 23·34 | 24·22 | 32·86 | 0·03 | 38,060 |
| 5–9 | 9·74 | 12·16 | 22·73 | 23·41 | 31·86 | 0·10 | 28,680 |
| 10–14 | 10·29 | 13·64 | 21·75 | 21·73 | 32·55 | 0·04 | 24,560 |
| 15–19 | 11·69 | 16·48 | 19·96 | 18·51 | 33·31 | 0·05 | 28,660 |
| 20–24 | 10·27 | 17·17 | 19·92 | 18·78 | 33·76 | 0·10 | 42,080 |
| 25–29 | 8·40 | 15·31 | 22·36 | 21·32 | 32·56 | 0·05 | 45,040 |
| 30–34 | 8·46 | 15·03 | 22·64 | 20·77 | 33·03 | 0·07 | 45,220 |
| 35–39 | 8·78 | 16·37 | 22·11 | 19·80 | 32·85 | 0·09 | 43,620 |
| 40–44 | 9·28 | 17·55 | 22·31 | 17·42 | 33·34 | 0·10 | 39,960 |
| 45–49 | 9·09 | 19·04 | 21·61 | 15·96 | 34·18 | 0·12 | 36,660 |
| 50–54 | 9·28 | 19·11 | 22·19 | 14·33 | 34·93 | 0·16 | 31,620 |
| 55–59 | 9·09 | 20·23 | 21·05 | 13·63 | 35·84 | 0·16 | 27,900 |
| 60–64 | 8·62 | 21·03 | 20·44 | 13·14 | 36·64 | 0·13 | 24,300 |
| 65–69 | 8·52 | 21·08 | 19·92 | 13·27 | 37·04 | 0·17 | 19,840 |
| 70–74 | 7·96 | 21·88 | 17·78 | 13·80 | 38·46 | 0·12 | 13,860 |
| 75–79 | 8·20 | 22·72 | 15·98 | 13·29 | 39·68 | 0·13 | 8,760 |
| 80–84 | 7·80 | 23·36 | 17·51 | 11·16 | 39·86 | 0·31 | 4,640 |
| 85–89 | 8·71 | 24·76 | 15·27 | 9·31 | 41·83 | 0·12 | 1,656 |
| 90–94 | 10·73 | 20·19 | 11·99 | 8·83 | 48·26 | — | 339 |
| 95– | — | 38·24 | 23·53 | 5·88 | 32·35 | — | 33 |
| Total | | | | | | | 505,488 |

incidence of cervical cancer in the population of rural areas shows about the same value—i.e., 50 per cent of the average for Copenhagen—as the wealthy borough of Gentofte (Table VII).

TABLE IV.—*Morbidity per* 10,000 *Living. Cancer of Various Sites. Copenhagen,* 1943 *to* 1947.

MALES.

| Age. | Stomach. | Oesophagus. | Prostate. | Lung. | Colon. | Sigmoideum. | Rectum. | All sites. |
|---|---|---|---|---|---|---|---|---|
| -24 | 0·0 | — | 0·0 | — | 0·0 | — | 0·1 | 1·4 |
| 25–29 | 0·1 | — | — | 0·1 | 0·1 | — | 0·2 | 3·1 |
| 30–34 | 0·3 | 0·1 | — | 0·2 | — | 0·1 | 0·2 | 3·4 |
| 35–39 | 0·6 | — | 0·1 | 0·5 | 0·4 | 0·2 | 0·4 | 5·8 |
| 40–44 | 1·8 | — | — | 2·6 | 0·4 | 0·2 | 0·8 | 11·0 |
| 45–49 | 1·7 | 0·5 | 0·1 | 4·9 | 0·6 | 0·5 | 1·9 | 18·6 |
| 50–54 | 4·8 | 0·6 | 0·6 | 7·4 | 1·1 | 1·1 | 3·7 | 31·7 |
| 55–59 | 7·2 | 0·7 | 2·1 | 14·7 | 3·3 | 1·5 | 5·5 | 55·6 |
| 60–64 | 11·1 | 3·7 | 5·0 | 13·2 | 5·7 | 2·3 | 9·9 | 81·8 |
| 65–69 | 19·8 | 6·0 | 8·9 | 7·9 | 6·3 | 5·6 | 14·2 | 108·2 |
| 70–74 | 30·9 | 10·3 | 17·1 | 10·5 | 10·1 | 6·1 | 19·2 | 160·8 |
| 75–79 | 44·4 | 12·3 | 23·9 | 13·1 | 11·6 | 10·1 | 25·7 | 214·2 |
| 80–84 | 57·3 | 14·5 | 23·1 | 6·8 | 16·2 | 5·1 | 17·1 | 217·1 |
| 85– | 77·1 | 11·7 | 21·0 | 4·7 | 16·4 | 9·3 | 23·4 | 278·0 |

FEMALES.

| Age. | Stomach. | Corpus uteri. | Breast. | Cervix uteri. | Colon. | Sigmoideum. | Rectum. | All sites. |
|---|---|---|---|---|---|---|---|---|
| -24 | — | — | 0·0 | 0·0 | 0·0 | — | — | 0·9 |
| 25–29 | 0·0 | — | 0·5 | 1·2 | 0·1 | 0·1 | 0·0 | 3·8 |
| 30–34 | 0·3 | — | 1·2 | 3·4 | 0·1 | 0·1 | 0·3 | 6·9 |
| 35–39 | 0·5 | 0·4 | 4·1 | 5·9 | 0·1 | 0·3 | 0·6 | 15·4 |
| 40–44 | 0·8 | 0·9 | 6·3 | 9·4 | 0·4 | 0·3 | 1·0 | 24·2 |
| 45–49 | 1·2 | 1·6 | 10·1 | 9·1 | 0·6 | 0·5 | 1·7 | 34·8 |
| 50–54 | 2·0 | 3·6 | 11·2 | 10·3 | 1·3 | 1·3 | 2·8 | 46·7 |
| 55–59 | 3·8 | 4·7 | 14·6 | 8·6 | 2·5 | 1·7 | 2·9 | 59·4 |
| 60–64 | 4·9 | 3·6 | 17·7 | 6·9 | 3·0 | 2·5 | 4·8 | 68·3 |
| 65–69 | 13·4 | 3·5 | 23·3 | 7·3 | 5·4 | 2·6 | 4·8 | 97·2 |
| 70–74 | 21·8 | 3·5 | 21·4 | 7·1 | 10·5 | 3·5 | 10·7 | 128·4 |
| 75–79 | 30·4 | 2·3 | 22·1 | 4·6 | 12·1 | 4·3 | 11·9 | 150·4 |
| 80–84 | 40·9 | 3·0 | 26·7 | 4·3 | 14·2 | 3·4 | 11·6 | 184·0 |
| 85– | 43·4 | 2·0 | 45·4 | 3·9 | 13·8 | 4·9 | 16·8 | 227·8 |

The assumption can be dismissed at once that a difference of this size may be due to less efficient notification of cases from rural areas. From these areas patients with cervical cancer are centralized for treatment in the Radiumstations of the Anti-Cancer League, situated in the towns of Copenhagen, Aarhus and Odense. The incidence of cervical cancer for provincial towns is intermediate between that for the capital and that for the rural areas.

Returning to the question about the influence of married life on the frequency of cervical cancer and the quotation opening this section, we have analyzed the variations in incidence of cervical cancer for the five districts separately for women never married, and for women married, widowed or divorced.

It will be evident that the range in variation of the ratio observed/computed for cervical cancer is wider for women never married than for women married, widowed or divorced.

The fact that there is a steeper gradient for the incidence of cervical cancer according to social class for women never married cannot be taken as any reliable indication either way of the influence of childbirth on the incidence of cervical cancer, since it is a justifiable assumption that the number of illegitimate children and of abortions is higher for the lower social level. Only studies of the social distribution of cervical cancer among childless women undertaken with due attention to their pregnancy histories will be able to produce a direct answer to this question.

The parallelism often found between the frequencies of births and of uterine cancer has suggested to some authors a causative relation between the two.

TABLE V.—*Distribution of Cancer Incidence in Copenhagen,* 1943–1947, *on Subdistricts of Various House Rent given as per cent of Standardized Average Value for Greater Copenhagen.*

| District. | | House rent, 1940. | Cervical cancer as per cent of computed value. | Female mammary cancer as per cent of computed value. | Male pulmonary cancer as per cent of average value. |
|---|---|---|---|---|---|
| I : 1440 Kr. | | | 50 | 93 | 66 |
| Gentofte | G | 1440 Kr. | 50 | 93 | 66 |
| II : 1050–1150 Kr. | | | 90 | 112 | 99 |
| Voldkvarterer | c | 1110 | 83 | 113 | 75 |
| Frederiksberg | F | 1055 | 92 | 112 | 107 |
| III : 850–950 Kr. | | | 79 | 110 | 90 |
| Østerbro | d | 910 | 82 | 115 | 95 |
| Emdrup | p | 910 | 124 | 67 | 69 |
| Vanløse | k | 895 | 92 | 114 | 94 |
| Vigerslev | j | 880 | 57 | 111 | 56 |
| Brønshøj | m | 855 | 69 | 96 | 101 |
| Amagerbro, etc. | r | 855 | 60 | 116 | 89 |
| IV : 750–850 Kr. | | | 100 | 94 | 97 |
| Sundbyøster S. | u | 820 | 83 | 105 | 86 |
| Husum | l | 810 | 106 | 63 | 68 |
| Valby | i | 805 | 94 | 112 | 109 |
| Sundbyvester | s | 790 | 119 | 95 | 110 |
| Kongens Enghave | h | 765 | 100 | 75 | 156 |
| Utterslev N | o | 765 | 104 | 80 | 72 |
| V : 645–750 Kr. | | | 131 | 92 | 116 |
| Utterslev S . | n | 730 | 115 | 108 | — |
| Old City | a | 710 | 154 | 101 | 120 |
| Outer Nørrebro | f | 675 | 117 | 85 | 119 |
| Sundbyøster N | t | 670 | 126 | 88 | 112 |
| Christianshavn | b | 655 | 190 | 134 | 92 |
| Vesterbro | g | 655 | 132 | 90 | 106 |
| Inner Nørrebro | e | 645 | 127 | 86 | 121 |

It would seem possible, however, that the social grading of the incidence of cervical cancer is governed not by births, but perhaps by pregnancies, in which case abortions could be reckoned to play their part. It should be noticed that the latter possibility allows the assumption of a part played by hormones in the aetiology of cervical cancer, while the former would tend to make mechanical injury a primary cause. Apart from the assumption that pregnancy has any

TABLE VIA.—*Cancer in Districts of Different Rental Groups Compared with Computed Standardized Values, Copenhagen, 1943–1947. Various Sites.*

MALES.

| District. | Stomach.* Observed. | Stomach.* Computed. | Stomach.* Obs./comp. (%). | Oesophagus.* Observed. | Oesophagus.* Computed. | Oesophagus.* Obs./comp. (%). | Prostate.* Observed. | Prostate.* Computed. | Prostate.* Obs./comp. (%). | Lung.** Observed. | Lung.** Computed. | Lung.** Obs./comp. (%). |
|---|---|---|---|---|---|---|---|---|---|---|---|---|
| I | 64 | 73·6 | 87 | 19 | 18·3 | 104 | 23 | 29·4 | 78 | 39 | 59·2 | 66 |
| II | 136 | 153·6 | 89 | 40 | 38·7 | 103 | 66 | 62·2 | 106 | 115 | 115·7 | 99 |
| III | 142 | 159·9 | 89 | 26 | 39·0 | 67 | 44 | 62·0 | 71 | 122 | 136·0 | 90 |
| IV | 148 | 124·4 | 119 | 24 | 30·1 | 80 | 51 | 47·8 | 107 | 102 | 105·2 | 97 |
| V | 338 | 314·4 | 108 | 96 | 78·4 | 122 | 144 | 125·9 | 114 | 285 | 245·7 | 116 |
| Unstated | — | 2·1 | — | — | 0·5 | — | — | 0·7 | — | 1 | 2·2 | — |

FEMALES.

| District. | Stomach.* Observed. | Stomach.* Computed. | Stomach.* Obs./comp. (%). | Corpus uteri.° Observed. | Corpus uteri.° Computed. | Corpus uteri.° Obs./comp. (%). | Breast.* Observed. | Breast.* Computed. | Breast.* Obs./comp. (%). | Cervix uteri.*** Observed. | Cervix uteri.*** Computed. | Cervix uteri.*** Obs./comp. (%). |
|---|---|---|---|---|---|---|---|---|---|---|---|---|
| I | 48 | 63·4 | 76 | 23 | 26·5 | 87 | 131 | 141·5 | 93 | 50 | 99·1 | 50 |
| II | 164 | 163·1 | 101 | 55 | 60·8 | 90 | 367 | 326·7 | 112 | 187 | 208·9 | 90 |
| III | 131 | 140·2 | 93 | 68 | 62·3 | 109 | 360 | 328·2 | 110 | 188 | 237·8 | 79 |
| IV | 104 | 100·1 | 104 | 46 | 42·7 | 108 | 220 | 233·8 | 94 | 179 | 178·5 | 100 |
| V | 308 | 287·0 | 107 | 109 | 108·3 | 101 | 541 | 586·5 | 92 | 509 | 387·3 | 131 |
| Unstated | — | 1·2 | — | — | 0·4 | — | — | 2·3 | — | — | 1·4 | — |

TABLE VIB.—*Cancer in Districts of Different Rental Groups Compared with Computed Standardized Values, Copenhagen,* 1943–1947. *Various Sites.*

MALES.

| District. | Colon.* Observed. | Colon.* Computed. | Colon.* Obs./comp. (%). | Sigmoideum.° Observed. | Sigmoideum.° Computed. | Sigmoideum.° Obs./comp. (%). | Rectum.° Observed. | Rectum.° Computed. | Rectum.° Obs./comp. (%). | All sites.*** Observed. | All sites.*** Computed. | All sites.*** Obs./comp. (%). |
|---|---|---|---|---|---|---|---|---|---|---|---|---|
| I | 17 | 25·2 | 67 | 21 | 15·6 | 135 | 42 | 49·6 | 85 | 393 | 451·3 | 87 |
| II | 66 | 51·5 | 128 | 31 | 32·1 | 97 | 101 | 100·9 | 100 | 933 | 917·2 | 102 |
| III | 41 | 54·6 | 75 | 37 | 34·0 | 109 | 115 | 108·6 | 106 | 913 | 1003·3 | 91 |
| IV | 40 | 42·2 | 95 | 28 | 26·6 | 105 | 87 | 84·6 | 103 | 800 | 790·0 | 101 |
| V | 115 | 105·8 | 109 | 58 | 66·2 | 88 | 209 | 208·7 | 100 | 2038 | 1906·5 | 107 |
| Unstated | 1 | 0·7 | — | — | 0·5 | — | — | 1·6 | — | 6 | 14·7 | — |

FEMALES.

| District. | Colon.° Observed. | Colon.° Computed. | Colon.° Obs./comp. (%). | Sigmoideum.* Observed. | Sigmoideum.* Computed. | Sigmoideum.* Obs./comp. (%). | Rectum.° Observed. | Rectum.° Computed. | Rectum.° Obs./comp. (%). | All sites.*** Observed. | All sites.*** Computed. | All sites.*** Obs./comp. (%). |
|---|---|---|---|---|---|---|---|---|---|---|---|---|
| I | 32 | 29·5 | 108 | 25 | 15·6 | 160 | 26 | 37·0 | 70 | 537 | 636·4 | 84 |
| II | 84 | 74·1 | 113 | 44 | 37·5 | 117 | 98 | 89·9 | 109 | 1577 | 1496·1 | 105 |
| III | 61 | 65·8 | 93 | 27 | 35·8 | 75 | 78 | 83·9 | 93 | 1420 | 1464·3 | 97 |
| IV | 39 | 47·0 | 83 | 18 | 25·3 | 71 | 60 | 59·9 | 100 | 1010 | 1054·5 | 96 |
| V | 131 | 131·1 | 100 | 67 | 66·6 | 101 | 169 | 159·7 | 106 | 2799 | 2683·2 | 104 |
| Unstated | 1 | 0·5 | — | — | 0·2 | — | — | 0·6 | — | 2 | 10·5 | — |

The statistical difference between the observed and computed distribution according to district are designated as follows for any single group of cancers:

° 5 per cent $< p$
* 1 per cent $< p <$ 5 per cent.
** 0·1 per cent $< p <$ 1 per cent.
*** $p <$ 0·1 per cent.

connection with the development of cancer, it should be pointed out that the occurrence of cervical cancer in virgins is questionable. Cases have been published of cervical cancer in children (Heckel, 1950), but these have been adenocarcinomata, so that it may be desirable in statistical work to separate this group from squamous cell cancers of the cervix. The present material is known to comprise 36 such cases, evenly distributed throughout the city.

*Mammary cancer.*

As recently discussed by one of us (Clemmesen, 1951), and as observed originally by Stern as early as 1844, there is an inverse relation between the frequency of uterine and mammary cancer. While the uterine cancer—that is, the cervical form—increases in frequency with marriage, mammary cancer, according to Stern (1844) will decrease in frequency with marriage. This inverse relationship tends to appear also in the present study, although not with invariable certainty. It will be seen that the wealthy District I is on the same low level as District V with regard to mammary cancer, although its birthrate is below the average for Greater Copenhagen (Clemmesen, 1951).

There is, however, a slight overall gradient opposite to the gradient for cervical cancer.

*Pulmonary cancer.*

It was demonstrated in a paper by Clemmesen and Busk (1947*c*) that even if lung carcinoma as a cause of death had tripled in the city of Copenhagen during the years 1936 to 1945, no corresponding increase was demonstrable in the data of the Central Tuberculosis Station for Copenhagen. There seemed to be a steep increase in the figures for those very years, when lung surgery and bronchoscopy were introduced. The authors concluded that no increase had been demonstrated which could not be explained through improvements of diagnostic facilities.

TABLE VII.—*Cancer Colli Uteri. Morbidity per* 10,000 *Living. Denmark,* 1943 *to* 1947.

| Age. | Copenhagen. | Provincial towns. | Rural areas. | Whole country. |
|---|---|---|---|---|
| –24 | 0·0 | 0·0 | 0·0 | 0·0 |
| 25–29 | 1·2 | 1·3 | 1·2 | 1·2 |
| 30–34 | 3·4 | 3·9 | 2·7 | 3·2 |
| 35–39 | 5·9 | 5·2 | 3·2 | 4·5 |
| 40–44 | 9·4 | 7·7 | 4·4 | 6·6 |
| 45–49 | 9·1 | 8·5 | 4·9 | 7·0 |
| 50–54 | 10·3 | 8·6 | 4·4 | 7·1 |
| 55–59 | 8·6 | 7·9 | 3·4 | 6·0 |
| 60–64 | 6·9 | 5·4 | 3·8 | 5·0 |
| 65–69 | 7·3 | 6·2 | 2·1 | 4·6 |
| 70–74 | 7·1 | 4·9 | 2·8 | 4·6 |
| 75–79 | 4·6 | 3·2 | 2·2 | 3·1 |
| 80–84 | 4·3 | 0·9 | 1·8 | 2·2 |
| 85– | 3·9 | 5·6 | 0·5 | 2·7 |

However, it appears from the histogram (Fig. 1) that male lung cancer shows an increase in frequency with lower social level. It is striking that the same irregularity appears for District II, as was the case for cervical cancer, for which site there is other evidence of a social difference in frequency.

*Gastric cancer.*

The decennial report of the English Registrar-General (1927, 1938) will be remembered as having demonstrated a marked social gradient for mortality from gastric cancer. A similar gradient is found in the present material, although its degree of significance does not lend any solid support to its reality, considering the difficulties involved in making this diagnosis. Contrary to the English findings there is no opposite tendency for intestinal cancers to occur with higher frequency in the upper classes.

### SUMMARY.

The incidence of various sites of cancer in Copenhagen for the years 1943 to 1947 is studied on the basis of the files of the Danish Cancer Registry. Cervical cancer shows a clear tendency to occur more often in the poorer classes both among married women and women never married. A similar tendency is demonstrated for cancer of the lung in males, while female mammary cancer shows a less pronounced tendency in the opposite direction.

The authors' thanks are due to the King Christian X Fund for a grant in support of the present study.

### REFERENCES.

Clemmesen, J.—(1950) *J. nat. Cancer, Inst.* **11**, 627.—(1951) *Ibid.* (in press).

*Idem* and Busk, T.—(1947*a*) *Cancer Res.*, **7**, 281.—(1947*b*) *Ibid.*, **7**, 286.—(1947*c*) *Brit. J. Cancer*, **1**, 253.—(1948*a*) *Cancer Res.*, **8**, 129.—(1948*b*) *Acta Radiol.*, **29**, 323.—(1948*c*) *Ibid.*, **30**, 9.—(1949*a*) *Cancer Res.*, **9**, 411.—(1949*b*) *Ibid.*, **9**, 415.

*Iidem* and Nielsen, A.—(1949) *Acta Radiol.*, **31**, 51.

Heckel, G. P.—(1950) *Pediatrics*, **5**, 924.

Registrar-General.—(1927) Decennial Supplement, England and Wales, 1921. Part II. London (H.M. Stationery Office.)—(1938) Decennial Supplement, England and Wales, 1931. Part II*a*. London (H.M. Stationery Office).

Stern, R.—(1842) *Giornali per Servire al Progressi Della Pathologia e della Terapeutica*, ser. 2, **2**, 507.—(1844) *Annali Universali di Medicina*, **110**, 484.